# How important is the land use mix measure in understanding walking behaviour? Results from the RESIDE study

**DOI:** 10.1186/1479-5868-8-55

**Published:** 2011-06-02

**Authors:** Hayley E Christian, Fiona C Bull, Nicholas J Middleton, Matthew W Knuiman, Mark L Divitini, Paula Hooper, Anura Amarasinghe, Billie Giles-Corti

**Affiliations:** 1 Centre for the Built Environment and Health, School of Population Health, The University of Western Australia, Crawley, Western Australia; 2 School of Population Health, The University of Western Australia, Crawley, Western Australia; 3 Formerly from School of Population Health, The University of Western Australia, Crawley, Western Australia

**Keywords:** Physical activity, Environment, Neighborhood, Walkability, Land use, Land use mix, Walking, Transport, Recreation, Entropy models, Methodology, Planning

## Abstract

**Background:**

Understanding the relationship between urban design and physical activity is a high priority. Different representations of land use diversity may impact the association between neighbourhood design and specific walking behaviours. This study examined different entropy based computations of land use mix (LUM) used in the development of walkability indices (WIs) and their association with walking behaviour.

**Methods:**

Participants in the RESIDential Environments project (RESIDE) self-reported mins/week of recreational, transport and total walking using the Neighbourhood Physical Activity Questionnaire (n = 1798). Land use categories were incrementally added to test five different LUM models to identify the strongest associations with recreational, transport and total walking. Logistic regression was used to analyse associations between WIs and walking behaviour using three cut points: any (> 0 mins), ≥ 60 mins and ≥ 150 mins walking/week.

**Results:**

Participants in high (vs. low) walkable neighbourhoods reported up to almost twice the amount of walking, irrespective of the LUM measure used. However, different computations of LUM were found to be relevant for different types and amounts of walking (i.e., > 0, ≥ 60 or ≥ 150 mins/week). Transport walking (≥ 60 mins/week) had the strongest and most significant association (OR = 2.24; 95% CI:1.58-3.18) with the WI when the LUM included 'residential', 'retail', 'office', 'health, welfare and community', *and *'entertainment, culture and recreation'. However, any (> 0 mins/week) recreational walking was more strongly associated with the WI (OR = 1.36; 95% CI:1.04-1.78) when land use categories included 'public open space', 'sporting infrastructure' and 'primary and rural' land uses. The observed associations were generally stronger for ≥ 60 mins/week compared with > 0 mins/week of transport walking and total walking but this relationship was not seen for recreational walking.

**Conclusions:**

Varying the combination of land uses in the LUM calculation of WIs affects the strength of relationships with different types (and amounts) of walking. Future research should examine the relationship between walkability and specific types and different amounts of walking. Our results provide an important first step towards developing a context-specific WI that is associated with recreational walking. Inherent problems with administrative data and the use of entropy formulas for the calculation of LUM highlight the need to explore alternative or complimentary measures of the environment.

## Background

Understanding the relationship between urban design and physical activity is now a high priority for the prevention of chronic disease [1,2]. It is estimated that by 2050, 70% of the forecasted world population of 9.1 billion will live in urban areas [3]. Identifying the specific characteristics of the urban environment that support or hinder people living an active lifestyle is important given the inadequate and declining levels of physical activity in both adults and children [4,5], increasing sedentary time related to electronic media use [6] and car travel times [7] and the rising level of obesity and non-communicable diseases [8,9].

Although the intention of many urban planning schemes implemented in the last 50 years has been to protect the public's health by separating industrial and residential areas to improve quality of life, emerging research suggests that these policies may have had unintended consequences [10,11]. Specifically, we may have inadvertently created residential environments that are detrimental to health because they are less supportive of physical activity, healthy eating and sustainable living [2,12-14]. As a consequence, there is a rapidly growing body of evidence investigating the relationship between attributes of the built environment and chronic disease risk factors.

Much of the published work addressing physical activity and the built environment has focused on composite walkability indices (WIs). The earliest work, undertaken by Frank et al., [15], used a WI with three sub-components (residential density; connectivity; and land use mix) and found significant associations with physical activity. Subsequent versions of the WI have modified the land use mix (LUM) computation by varying or adding new categories (e.g., adding retail floor area ratio) [16,17]. Studies of the association between the WI and physical activity have been replicated in Australia [18-20] and elsewhere [21,22]. However, in attempting to replicate Frank and colleague's original WI, modifications are often required due to differences in both the structure and availability of secondary data for the study area. Nonetheless, overall the literature suggests a positive association between WIs and walking for transport [19]. Notably, no consistent relationship has been shown between WIs and patterns of recreational walking, and results also appear mixed for total walking [12]. Consequently, there has been a call for more robust measures and a matching of the built environment measure to the behaviour of interest and at the appropriate scale (i.e., context-specific measures) [23-26]. Notably, much of the research has been conducted in North America and further investigation of these relationships are required in countries (and cities) with varying urban, cultural and demographic environments [24,27], and using different data sources.

Often insufficient methodological detail is reported on the computation of the WI and its sub-components and as a result this has prevented a more detailed analysis and comparison between studies [28,29]. One particular area where more transparency is needed is in the measurement and computation of LUM variables. At present there is no conclusive evidence on what aspects of land use are most important to encourage different types of walking and physical activity. The apparent inconsistent findings may be due to differences in the measurement methods of the built environment and physical activity [29-31]. Currently, the association between LUM and physical activity is assessed most often using entropy models [12,32] which represent the extent of variation (or mix) in the distribution of land uses. Land use categories that may support or hinder physical activity are selected for inclusion. There is, however, a need to better understand how different representations of land use diversity impact on the association between neighbourhood design and specific walking behaviours (i.e., transport or recreational) and whether varying the composition and/or combination of land use classifications impacts on these associations. Therefore, the aim of this paper was to examine different entropy based computations of LUM used in the development of WIs and their association with walking behaviour.

## Methods

Baseline data (n = 1798; 15 had missing environmental data) from the RESIDential Environments project (RESIDE) was used for this study. RESIDE is a quasi-experimental longitudinal study evaluating the impact of the Western Australian government's new sub-division design code on walking, cycling, public transport use and sense of community. A detailed description of RESIDE's study design and sampling procedures is published elsewhere [33]. Briefly, the study involved a cohort of people (n = 1813) building new homes in 74 new housing developments (18 of which were designed using the new design code), who were initially surveyed three times: before moving into their new home (baseline), and 12 and 36 months after. The baseline data was collected before people moved into their new home and thus participants were distributed throughout the Perth metropolitan area (500 square kilometres and a population of 1.7 million).

### Self-reported physical activity

Physical activity was measured using the Neighbourhood Physical Activity Questionnaire (NPAQ) [34]. NPAQ records participants' walking behaviour within and outside the neighbourhood and has acceptable reliability [34]. NPAQ defines the neighbourhood as a 10-15 minute (1600 m) walk from a participant's home because it represents approximately how far a participant could walk from their house at moderate to vigorous intensity pace within 15 minutes, half the recommended level of daily physical activity for adults [35]. Participants were asked whether in a usual week they walked within their local neighbourhood for recreation, health or fitness (classified as walking for recreation) or to get to or from somewhere (classified as walking for transport). In this study, dichotomous variables (yes/no) were computed for > 0 (any), ≥ 60 and ≥ 150 mins/week of walking for recreation and walking for transport in the neighbourhood. Total minutes of walking for those who did some walking were also dichotomised (yes/no) at > 0, ≥ 60 and ≥ 150 mins/week.

### Walkability Indice

Three design characteristics were used to construct a WI used to represent each participant's residential neighbourhood: Street connectivity; Net residential density; and LUM. A WI score for each participant was calculated at the walkable service area level (defined as a 15 minute walk (1.6 km) street network buffer) by summing the standard z-scores of the three attributes. The WI score was quartiled, grouping residents into Low, Medium/Low, Medium/High and High walkable environments.

Street connectivity measures the inter-connectedness of the street network within a participant's walkable service area. The measure is a ratio of the count of three (or more) way intersections over the area (km^2^). Net residential density measures the density of dwellings on residential land within a participant's service area. The formula is a ratio of the number of residential dwellings over the area in residential use (in hectares). Both street connectivity and residential density measures were based on methods used by Frank et al., [15]. The formula used to calculate LUM was a variation of the entropy formula also used by Frank et al., [15,36].(1)

Where H = land use mix score, *pi *= the proportion of the area covered by land use *i *against the summed area for land use classes of interest (including *i*), and *n *is the number of land use classes of interest. Land use classifications were obtained from two sources: land tenure (taxation/rating) records (Valuer General's Office) [37] and reserve vesting information [38]. Records from these sources were re-coded to a modified set of the Planning Land Use Classes defined by the Ministry for Planning Western Australia [39]. Land use was allocated to cadastral parcels [40] on a mutually exclusive basis (with all overlaps eliminated), based on a hierarchy of preference (see Table 1).

**Table 1:** Classification hierarchy of planning land use classes based on Western Australian Ministry for Planning (2000)

1	Shop/Retail
2	Other Retail
3	Office/Business
4	Health/Welfare & Community Services
5	Entertainment/Recreational & Cultural
51	Public Open Space
52	Sporting infrastructure
6	Primary/Rural (note this includes extractive industries, farming and conservation areas)
7	Manufacturing/Processing/Fabrication
8	Storage & Distribution
9	Service Industry
10	Residential
11	Utilities/Communications
12	Vacant Floor Area
13	Vacant Land Area
0	Unclassified land (areas lacking any land use data)

Given the focus of this paper was to test five different models of LUM, we undertook a review of the literature to assess land use classes used in WIs by others in order to build models that best fit with total walking, recreational walking and transport walking. Land use classes were incrementally added to a base LUM model. The computation for each LUM is outlined in Table 2. Models 1 to 4 included 'Residential', 'Retail', 'Office' and 'Health, welfare and community' land use classes. Model 1 reflects the early work by Frank and is the simplest land use computation [15]. Model 2 adds 'Entertainment, culture and recreation' into the LUM thus capturing built recreational destinations and reflecting later models used by Frank et al., [16,17]. Model 3 was the same as model 2 with the addition of the 'Public open space, sporting infrastructure and primary and rural' land use classes as used by Forsyth et al., [41]. These land classes may be relevant for recreational walking and physical activity directly (e.g., sports facilities) or indirectly (e.g., rural areas). Model 4 extended Model 3 with the addition of 'Unclassified land' because in Western Australia these land uses include some types of facilities (e.g., cultural and public service) and/or undeveloped areas untaxed by the state (e.g., natural landscapes) that may provide some amenity for walking. Model 5 was based on model 4 however it excluded 'Unclassified land' and 'Office' land use classes and represents our attempt to develop a model to better explain patterns of recreational walking.

**Table 2:** Summary of land use classes used in five models of Land Use Mix

**Land use class**^**a**^	Model 1	Model 2	Model 3	Model 4	Model 5
Retail (1)	*	*	*	*	*
Office (3)	*	*	*	*	**-**
Health, welfare & community (4)	*	*	*	*	*
Entertainment, culture & recreation (5)	**-**	*	*	*	*
Public open space, Sporting infrastructure & Primary and rural (51+52+6)	**-**	**-**	*	*	*
Residential (10)	*	*	*	*	*
Unclassified (0)	**-**	**-**	**-**	*	**-**

^a ^Planning land use classes included from Table 1

All Geographic Information Systems (GIS) analyses were undertaken using Environmental System Research Institutes, ArcGIS 9.2 Desktop software [42]. Service areas (1.6 km) were generated using the Network Analyst extension in ArcGIS, using settings that "trimmed" service areas to a maximum distance of 100 m from road segments.

### Statistical analysis

Logistic regression was used to analyse the association between WIs and total walking, transport walking and recreational walking in the neighbourhood by three cut points, > 0, ≥ 60 and ≥ 150 mins/week. The WI z-score was considered in its original continuous form, in quartiles and as a trend across quartiles. Results from the logistic regression models are presented as estimated odds ratios (OR), with the lowest WI quartile used as the reference level. Models were also fitted simultaneously including the three individual components of the z-score; LUM, net residential density and street connectivity. All regression models adjusted for gender, age, education level, marital status and presence of children at home. All analyses were carried out using Proc Logistic in SAS Version 9.2.

## Results

At baseline, the mean age of RESIDE participants was 40 years (range 19-78) and 60% were female. On average, participants did 94 minutes of total walking/week within the neighbourhood, comprising an average of 68 minutes of recreational walking and 26 minutes of transport-related walking (Table 3). By construction, the mean (Standard Deviation (SD)) z-score for residential density, street connectivity and LUM were 0.00 (1.00) and 0.00 (2.17) for the WI (Table 3).

**Table 3:** Sample and neighbourhood characteristics

	n = 1,798
**Sample characteristic**	
Gender female (%)	59.5
Mean age	39.9 (11.9)
Mean minutes of total neighbourhood walking	93.5 (122.9)
> 0 (%)	61.6
≥ 60 mins/week (%)	49.7
≥ 150 mins/week (%)	26.1
Mean minutes of neighbourhood walking for transport	26.0 (56.7)
> 0 (%)	35.9
≥ 60 mins/week (%)	17.3
≥ 150 mins/week (%)	4.1
Mean minutes of neighbourhood walking for recreation	67.5 (98.0)
> 0 (%)	52.5
≥ 60 mins/week (%)	42.0
≥ 150 mins/week (%)	18.4
**Neighbourhood characteristics**	
Z-score residential density	0.00 (1.00)
Z-score connectivity	-0.00 (1.00)
Z-score land use mix (Model 1)	0.00 (1.00)
Z-score walkability index (Model 1)	0.00 (2.17)

### Association between WI and any (> 0 mins/week) walking

Model 1 (Table 4) includes the WI with LUM computed with the least number of land uses. The odds of doing any walking for transport was 1.81 times higher for participants living in high walkable neighbourhoods and about 1.3 times higher for participants living in medium-low and medium-high walkable neighbourhoods, compared with low walkable neighbourhoods. The trend test was statistically significant (p < 0.01). The association between the WI and any recreational or any walking overall was not significant in Model 1. The trend test for the continuous measure of the WI reached statistical significance only for transport walking and total walking (p ≤ 0.01).

**Table 4:** Association between neighbourhood walkability and any (> 0 mins/week) walking (transport, recreation and total) using different computations of land use mix

	**Model 1**^**a**^		**Model 2**^**b**^		**Model 3**^**c**^		**Model 4**^**d**^		**Model 5**^**e**^	
WI Quartile	OR (95% CI)	p-value	OR (95% CI)	p-value	OR (95% CI)	p-value	OR (95% CI)	p-value	OR (95% CI)	p-value
**Do any transport walking (> 0 mins/week):**										
Low	1.00		1.00		1.00		1.00		1.00	
Med-Low	1.34 (1.01,1.78)	**0.040**	1.49 (1.13,1.98)	**< 0.01**	1.37 (1.04,1.82)	**0.028**	1.15 (0.87,1.51)	0.333	1.42 (1.08,1.88)	**0.014**
Med-High	1.26 (0.95,1.67)	0.112	1.26 (0.94,1.67)	0.117	1.33 (1.00,1.76)	0.051	0.98 (0.74,1.29)	0.875	1.26 (0.95,1.67)	0.109
High	1.81 (1.37,2.39)	**< 0.01**	1.96 (1.48,2.59)	**< 0.01**	1.58 (1.20,2.09)	**< 0.01**	1.28 (0.97,1.68)	0.078	1.53 (1.16,2.03)	**< 0.01**
Trend (quartiles)	1.19 (1.09,1.30)	**< 0.01**	1.20 (1.10,1.31)	**< 0.01**	1.14 (1.05,1.25)	**< 0.01**	1.06 (0.97,1.16)	0.183	1.12 (1.03,1.22)	**0.011**
Trend (z-score)	1.12 (1.07,1.18)	**< 0.01**	1.13 (1.08,1.18)	**< 0.01**	1.10 (1.05,1.16)	**< 0.01**	1.09 (1.04,1.14)	**< 0.01**	1.10 (1.05,1.15)	**< 0.01**
										
Residential density (z-score)*	1.10 (0.99,1.24)	0.080	1.10 (0.98,1.23)	0.092	1.14 (1.02,1.27)	**0.023**	1.15 (1.03,1.28)	**0.012**	1.14 (1.02,1.27)	**0.019**
Connectivity (z-score)*	1.14 (1.03,1.27)	**0.014**	1.15 (1.03,1.28)	**0.014**	1.14 (1.03,1.27)	**0.016**	1.15 (1.03,1.28)	**0.013**	1.14 (1.02,1.27)	**0.017**
Land use mix (z-score)*	1.13 (1.02,1.25)	**0.021**	1.15 (1.03,1.27)	**0.010**	1.03 (0.93,1.14)	0.575	0.97 (0.88,1.07)	0.541	1.02 (0.92,1.13)	0.726
**Do any recreational walking (> 0 mins/week):**										
Low	1.00		1.00		1.00		1.00		1.00	
Med-Low	0.92 (0.71,1.20)	0.543	0.99 (0.76,1.29)	0.935	1.04 (0.80,1.36)	0.771	0.84 (0.65,1.10)	0.203	0.88 (0.67,1.14)	0.336
Med-High	0.94 (0.72,1.23)	0.648	0.97 (0.74,1.26)	0.808	1.05 (0.81,1.37)	0.713	1.01 (0.78,1.32)	0.922	1.00 (0.77,1.30)	0.995
High	1.15 (0.88,1.50)	0.319	1.17 (0.89,1.52)	0.263	1.36 (1.04,1.78)	**0.024**	1.25 (0.96,1.64)	0.101	1.29 (0.99,1.69)	0.061
Trend (quartiles)	1.04 (0.96,1.14)	0.323	1.04 (0.96,1.14)	0.314	1.10 (1.01,1.19)	**0.030**	1.09 (1.00,1.18)	**0.049**	1.09 (1.01,1.19)	**0.038**
Trend (z-score)	1.02 (0.98,1.07)	0.294	1.02 (0.98,1.07)	0.343	1.03 (0.98,1.07)	0.231	1.04 (1.00,1.09)	0.062	1.03 (0.98,1.07)	0.216
										
Residential density (z-score)*	0.99 (0.89,1.11)	0.893	1.00 (0.89,1.11)	0.943	0.99 (0.89,1.10)	0.868	0.98 (0.88,1.09)	0.741	0.99 (0.89,1.10)	0.890
Connectivity (z-score)*	1.06 (0.95,1.17)	0.301	1.06 (0.95,1.17)	0.300	1.05 (0.95,1.17)	0.319	1.05 (0.95,1.16)	0.367	1.05 (0.95,1.17)	0.347
Land use mix (z-score)*	1.03 (0.93,1.13)	0.599	1.02 (0.92,1.12)	0.765	1.04 (0.94,1.15)	0.415	1.12 (1.01,1.23)	**0.025**	1.04 (0.95,1.15)	0.387
**Do any walking at all (> 0 mins/week):**										
Low	1.00		1.00		1.00		1.00		1.00	
Med-Low	0.96 (0.73, 1.26)	0.765	1.04 (0.80,1.37)	0.759	1.08 (0.83,1.42)	0.571	0.91 (0.69,1.19)	0.491	1.07 (0.82,1.40)	0.618
Med-High	1.00 (0.76, 1.32)	0.980	1.05 (0.80,1.37)	0.738	1.10 (0.84,1.44)	0.506	0.93 (0.71,1.23)	0.625	1.03 (0.79,1.35)	0.832
High	1.28 (0.97, 1.69)	0.078	1.33 (1.01,1.75)	**0.043**	1.35 (1.03,1.78)	**0.032**	1.20 (0.91,1.59)	0.187	1.34 (1.02,1.76)	**0.038**
Trend (quartiles)	1.08 (0.99, 1.18)	0.080	1.09 (1.00,1.19)	0.056	1.10 (1.00,1.19)	**0.040**	1.06 (0.97,1.15)	0.197	1.09 (1.00,1.18)	0.063
Trend (z-score)	1.06 (1.01, 1.11)	**0.010**	1.06 (1.01,1.11)	**0.011**	1.06 (1.01,1.11)	**0.017**	1.07 (1.02,1.12)	**< 0.01**	1.06 (1.01,1.11)	**0.022**
										
Residential density (z-score)*	1.03 (0.92,1.16)	0.567	1.04 (0.92,1.16)	0.550	1.05 (0.93,1.18)	0.424	1.05 (0.94,1.18)	0.404	1.05 (0.94,1.18)	0.368
Connectivity (z-score)*	1.06 (0.95,1.18)	0.278	1.06 (0.95,1.18)	0.273	1.06 (0.95,1.18)	0.307	1.06 (0.95,1.18)	0.328	1.05 (0.95,1.17)	0.338
Land use mix (z-score)*	1.10 (0.99,1.22)	0.069	1.10 (0.99,1.22)	0.083	1.07 (0.97,1.19)	0.183	1.10 (0.99,1.21)	0.067	1.06 (0.96,1.17)	0.265

OR = odds ratio; CI = confidence interval; *Denotes multivariate association; ^a ^Model 1 = Residential+Retail+Office+Health, welfare & community; ^b ^Model 2 = Model 1+Entertainment, culture & recreation; ^c ^Model 3 = Model 2+Public Open Space, Sporting infrastructure & Primary and rural; ^d ^Model 4 = Model 3+Unclassified land; ^e ^Model 5 = Residential+Retail+Health, welfare & community+Entertainment, culture & recreation+Public open space, Sporting infrastructure & Primary and rural. All models adjusted for gender, age, education level, marital status and presence of children at home.

The addition of the 'Entertainment, culture and recreation' land use category in the WI used in Model 2 (Table 4) resulted in slightly stronger effect sizes for the association between the WI and doing any transport walking and this was reflected in the association with total walking also. The odds of walking for transport was 1.96 times higher for participants living in high walkable neighbourhoods (p < 0.01) and 1.49 and 1.26 times higher for participants living in medium-low and medium-high walkable neighbourhoods, respectively, compared with low walkable neighbourhoods. The test for trend was also significant for both categorical and continuous variables (p < 0.01). Once again, the WI in Model 2 was not significantly associated with doing any recreational walking. However, the odds of total walking was 1.33 times higher for participants living in high compared with low walkable neighbourhoods (slightly larger than the odds of 1.28 seen in Model 1) (p < 0.05).

'Public open space', 'Sporting infrastructure' and 'Primary and rural' land use categories were added in Model 3 (Table 4). However, this reduced the effect size of the association between the WI and doing any transport walking and increased the association with doing any recreational walking. The odds of walking for recreation was 1.36 times higher for participants living in high compared with low walkable neighbourhoods (p < 0.05). The odds of walking for transport reduced to 1.58 for participants living in high walkable neighbourhoods compared with low walkable neighbourhoods. The odds of doing any walking at all increased slightly to 1.35 for participants living in high compared with low walkable neighbourhoods and both trend tests were statistically significant (p < 0.05).

Adding 'Unclassified' land into the LUM measure in Model 4 resulted in a significant reduction in effect sizes for the association between WIs and doing all types of walking (total, recreational and transport). However, some trend tests remained significant (Table 4).

Based on observations from Model 3 and 4, Model 5 was our attempt to improve the association between the WI and recreational walking (Table 4). However, Model 5 was no better than Model 3 for recreational walking. Moreover, the results for transport walking (OR = 1.53) and total walking (OR = 1.34) were comparable to Model 3.

### Association between components of the WI and any (> 0 mins/week) walking

When the three components of the WI z-score (LUM, residential density and connectivity) were modelled separately instead of as an overall WI, only LUM was significantly associated with recreational walking and only in Model 4. For transport walking, connectivity was significant in all Models, and LUM was significant in Models 1 and 2 but not in Models 3, 4 and 5 after including 'Public open space', 'Sporting infrastructure' and 'Primary and rural' land uses. Residential density approached significance in Models 1 and 2 and was significant in Models 3, 4 and 5 for transport walking. None of the components were significant in any of the models for total walking.

### Association between WI and ≥ 60 mins/week and ≥ 150 mins/week of walking

We also explored the association between WIs and the *amount *of walking reported and used two cut points, namely ≥ 60 mins/week (Table 5) and ≥ 150 mins/week (Table 6). Overall, the relationship between WIs and ≥ 60 mins/week transport walking was similar to the results for any transport walking (> 0 mins/week) (Table 5). However, the effect sizes across all five models were generally stronger for ≥ 60 mins/week transport walking and total walking compared with any (> 0 mins/week) walking. In contrast, no significant associations were found for ≥ 60 mins/week recreational walking except for a modest categorical trend test in Model 4.

**Table 5:** Association between neighbourhood walkability and ≥ 60 mins/week of walking (transport, recreation and total) using different computations of land use mix

	**Model 1**^**a**^		**Model 2**^**b**^		**Model 3**^**c**^		**Model 4**^**d**^		**Model 5**^**e**^	
WI Quartile	OR (95% CI)	p-value	OR (95% CI)	p-value	OR (95% CI)	p-value	OR (95% CI)	p-value	OR (95% CI)	p-value
**Transport walking ≥ 60 min/week:**										
Low	1.00		1.00		1.00		1.00		1.00	
Med-Low	1.32 (0.91,1.90)	0.143	1.42 (0.98,2.05)	0.064	1.40 (0.97,2.02)	0.071	0.77 (0.53,1.13)	0.184	1.31 (0.91,1.89)	0.151
Med-High	1.06 (0.72,1.54)	0.779	1.00 (0.68,1.48)	0.984	1.29 (0.89,1.87)	0.187	1.25 (0.88,1.78)	0.204	1.29 (0.89,1.86)	0.181
High	2.02 (1.43,2.87)	**< 0.01**	2.24 (1.58,3.18)	**< 0.01**	1.78 (1.25,2.55)	**< 0.01**	1.47 (1.05,2.07)	**0.026**	1.77 (1.24,2.52)	**< 0.01**
Trend (quartiles)	1.23 (1.10,1.37)	**< 0.01**	1.25 (1.12,1.40)	**< 0.01**	1.18 (1.05,1.32)	**< 0.01**	1.18 (1.06,1.32)	**< 0.01**	1.19 (1.06,1.33)	**< 0.01**
Trend (z-score)	1.11 (1.06,1.17)	**< 0.01**	1.12 (1.06,1.18)	**< 0.01**	1.11 (1.05,1.17)	**< 0.01**	1.11 (1.05,1.17)	**< 0.01**	1.10 (1.04,1.16)	**< 0.01**
Residential density (z-score)*	1.03 (0.91,1.17)	0.635	1.03 (0.90,1.17)	0.689	1.05 (0.93,1.19)	0.438	1.05 (0.93,1.19)	0.432	1.06 (0.94,1.20)	0.352
Connectivity (z-score)*	1.22 (1.07,1.39)	**< 0.01**	1.22 (1.07,1.39)	**< 0.01**	1.21 (1.07,1.38)	**< 0.01**	1.21 (1.06,1.38)	**< 0.01**	1.21 (1.06,1.38)	**< 0.01**
Land use mix (z-score)*	1.12 (0.99,1.28)	0.068	1.14 (1.01,1.29)	**0.037**	1.07 (0.95,1.21)	0.282	1.09 (0.97,1.24)	0.156	1.04 (0.92,1.18)	0.537
**Recreation walking ≥ 60 min/week:**										
Low	1.00		1.00		1.00		1.00		1.00	
Med-Low	0.91 (0.70,1.19)	0.502	0.91 (0.69,1.19)	0.472	0.98 (0.75,1.28)	0.866	0.96 (0.73,1.26)	0.757	0.86 (0.66,1.13)	0.285
Med-High	0.86 (0.66,1.13)	0.278	0.85 (0.65,1.11)	0.241	0.99 (0.75,1.30)	0.937	1.09 (0.83,1.42)	0.540	1.03 (0.78,1.34)	0.849
High	1.10 (0.84,1.44)	0.482	1.12 (0.86,1.46)	0.412	1.29 (0.99,1.69)	0.062	1.29 (0.98,1.68)	0.065	1.24 (0.95,1.63)	0.110
Trend (quartiles)	1.02 (0.94,1.11)	0.591	1.03 (0.94,1.12)	0.525	1.08 (0.99,1.18)	0.071	1.09 (1.00,1.19)	**0.041**	1.09 (1.00,1.18)	0.054
Trend (z-score)	1.02 (0.97,1.06)	0.496	1.01 (0.97,1.06)	0.529	1.02 (0.98,1.07)	0.309	1.04 (0.99,1.09)	0.085	1.03 (0.98,1.07)	0.254
Residential density (z-score)*	1.03 (0.93,1.15)	0.560	1.03 (0.93,1.15)	0.539	1.02 (0.92,1.14)	0.689	1.01 (0.91,1.12)	0.850	1.02 (0.92,1.13)	0.708
Connectivity (z-score)*	1.03 (0.93,1.14)	0.594	1.03 (0.93,1.14)	0.595	1.03 (0.93,1.14)	0.610	1.02 (0.92,1.13)	0.684	1.02 (0.92,1.14)	0.648
Land use mix (z-score)*	0.98 (0.89,1.08)	0.708	0.98 (0.88,1.08)	0.625	1.02 (0.92,1.13)	0.696	1.10 (1.00,1.21)	0.054	1.03 (0.94,1.14)	0.509
**Total walking ≥ 60 min/week:**										
Low	1.00		1.00		1.00		1.00		1.00	
Med-Low	0.96 (0.74,1.25)	0.774	1.00 (0.77,1.30)	0.991	1.14 (0.87,1.48)	0.347	0.92 (0.70,1.20)	0.530	0.94 (0.72,1.23)	0.651
Med-High	0.94 (0.72,1.22)	0.625	0.92 (0.71,1.20)	0.544	1.07 (0.82,1.40)	0.604	1.08 (0.83,1.41)	0.556	1.03 (0.79,1.34)	0.828
High	1.35 (1.04,1.77)	**0.026**	1.38 (1.05,1.79)	**0.019**	1.48 (1.14,1.94)	**< 0.01**	1.36 (1.05,1.78)	**0.022**	1.39 (1.06,1.81)	**0.016**
Trend (quartiles)	1.09 (1.00,1.19)	**0.041**	1.09 (1.00,1.19)	**0.043**	1.12 (1.03,1.22)	**< 0.01**	1.12 (1.03,1.21)	**0.011**	1.11 (1.02,1.21)	**0.012**
Trend (z-score)	1.06 (1.01,1.11)	**0.011**	1.06 (1.01,1.11)	**0.011**	1.06 (1.01,1.11)	**0.010**	1.08 (1.03,1.13)	**< 0.01**	1.06 (1.01,1.11)	**0.011**
Residential density (z-score)*	1.04 (0.93,1.15)	0.525	1.04 (0.93,1.15)	0.524	1.04 (0.93,1.16)	0.476	1.04 (0.93,1.15)	0.526	1.04 (0.94,1.16)	0.422
Connectivity (z-score)*	1.08 (0.97,1.20)	0.156	1.08 (0.97,1.20)	0.154	1.07 (0.97,1.19)	0.174	1.07 (0.96,1.19)	0.204	1.07 (0.97,1.19)	0.197
Land use mix (z-score)*	1.07 (0.97,1.18)	0.202	1.07 (0.97,1.18)	0.202	1.07 (0.97,1.18)	0.179	1.13 (1.03,1.25)	**0.011**	1.06 (0.96,1.17)	0.230

OR = odds ratio; CI = confidence interval; *Denotes multivariate association; ^a ^Model 1 = Residential+Retail+Office+Health, welfare & community; ^b ^Model 2 = Model 1+Entertainment, culture & recreation; ^c ^Model 3 = Model 2+Public Open Space, Sporting infrastructure & Primary and rural; ^d ^Model 4 = Model 3+Unclassified land; ^e ^Model 5 = Residential+Retail+Health, welfare & community+Entertainment, culture & recreation+Public open space, Sporting infrastructure & Primary and rural. All models adjusted for gender, age, education level, marital status and presence of children at home.

**Table 6:** Association between neighbourhood walkability and ≥ 150 mins/week of walking (transport, recreation and total) using different computations of land mix use

	**Model 1**^**a**^		**Model 2**^**b**^		**Model 3**^**c**^		**Model 4**^**d**^		**Model 5**^**e**^	
WI Quartile	OR (95% CI)	p-value	OR (95% CI)	p-value	OR (95% CI)	p-value	OR (95% CI)	p-value	OR (95% CI)	p-value
**Transport walking ≥ 150 min/week:**										
Low	1.00		1.00		1.00		1.00		1.00	
Med-Low	0.84 (0.42,1.70)	0.637	0.85 (0.42,1.72)	0.652	1.93 (0.96,3.85)	0.064	0.42 (0.19,0.94)	**0.034**	1.47 (0.75,2.88)	0.266
Med-High	0.73 (0.35,1.52)	0.402	0.74 (0.36,1.54)	0.420	1.06 (0.48,2.33)	0.883	1.26 (0.68,2.34)	0.453	1.08 (0.53,2.23)	0.827
High	1.58 (0.86,2.93)	0.144	1.60 (0.86,2.96)	0.134	1.90 (0.95,3.81)	0.070	1.03 (0.55,1.94)	0.933	1.41 (0.71,2.79)	0.325
Trend (quartiles)	1.17 (0.94,1.44)	0.152	1.17 (0.95,1.45)	0.143	1.14 (0.92,1.40)	0.232	1.11 (0.90,1.37)	0.343	1.07 (0.87,1.32)	0.527
Trend (z-score)	1.12 (1.02,1.22)	**0.016**	1.12 (1.03,1.23)	**0.012**	1.09 (1.00,1.20)	0.062	1.10 (1.00,1.21)	**0.040**	1.08 (0.98,1.19)	0.122
Residential density (z-score)*	1.12 (0.90,1.39)	0.313	1.11 (0.89,1.38)	0.352	1.16 (0.94,1.43)	0.163	1.14 (0.93,1.41)	0.215	1.18 (0.96,1.45)	0.126
Connectivity (z-score)*	1.26 (1.00,1.60)	0.052	1.26 (1.00,1.60)	0.053	1.28 (1.01,1.62)	**0.039**	1.29 (1.01,1.64)	**0.040**	1.30 (1.03,1.64)	**0.027**
Land use mix (z-score)*	0.99 (0.78,1.25)	0.909	1.01 (0.80,1.28)	0.922	0.85 (0.66,1.09)	0.198	0.89 (0.70,1.13)	0.341	0.78 (0.60,1.00)	**0.047**
**Recreation walking ≥ 150 min/week:**										
Low	1.00		1.00		1.00		1.00		1.00	
Med-Low	0.83 (0.59,1.17)	0.290	0.84 (0.60,1.18)	0.311	1.09 (0.77,1.53)	0.632	0.96 (0.68,1.36)	0.817	0.83 (0.59,1.19)	0.313
Med-High	0.84 (0.60,1.19)	0.324	0.78 (0.55,1.11)	0.169	0.83 (0.58,1.19)	0.319	0.97 (0.68,1.37)	0.846	0.98 (0.69,1.38)	0.901
High	0.94 (0.67,1.33)	0.742	0.97 (0.69,1.36)	0.842	1.16 (0.82,1.63)	0.401	1.08 (0.77,1.53)	0.644	1.08 (0.77,1.51)	0.664
Trend (quartiles)	0.98 (0.88,1.10)	0.747	0.98 (0.88,1.10)	0.738	1.02 (0.91,1.14)	0.729	1.03 (0.92,1.14)	0.652	1.04 (0.93,1.16)	0.494
Trend (z-score)	1.01 (0.96,1.07)	0.665	1.01 (0.96,1.07)	0.669	1.01 (0.96,1.07)	0.616	1.02 (0.96,1.08)	0.526	1.02 (0.96,1.08)	0.503
Residential density (z-score)*	1.00 (0.86,1.15)	0.964	1.00 (0.86,1.15)	0.967	1.00 (0.86,1.15)	0.955	1.00 (0.86,1.15)	0.947	0.99 (0.86,1.14)	0.917
Connectivity (z-score)*	1.02 (0.89,1.17)	0.771	1.02 (0.89,1.17)	0.769	1.02 (0.89,1.17)	0.787	1.02 (0.89,1.17)	0.807	1.01 (0.88,1.16)	0.836
Land use mix (z-score)*	1.02 (0.90,1.16)	0.727	1.02 (0.90,1.16)	0.737	1.03 (0.91,1.17)	0.626	1.05 (0.93,1.19)	0.451	1.06 (0.93,1.20)	0.395
**Total walking ≥ 150 min/week:**										
Low	1.00		1.00		1.00		1.00		1.00	
Med-Low	0.85 (0.62,1.15)	0.286	0.86 (0.63,1.16)	0.316	1.16 (0.85,1.57)	0.344	0.88 (0.65,1.20)	0.417	0.98 (0.72,1.33)	0.873
Med-High	0.84 (0.62,1.14)	0.268	0.79 (0.58,1.08)	0.136	0.93 (0.68,1.27)	0.628	1.06 (0.78,1.44)	0.715	1.01 (0.74,1.37)	0.958
High	1.03 (0.76,1.39)	0.852	1.11 (0.83,1.50)	0.483	1.27 (0.94,1.72)	0.118	1.15 (0.85,1.55)	0.365	1.19 (0.88,1.61)	0.262
Trend (quartiles)	1.01 (0.91,1.11)	0.881	1.03 (0.93,1.13)	0.609	1.05 (0.96,1.16)	0.292	1.06 (0.96,1.17)	0.219	1.06 (0.96,1.16)	0.254
Trend (z-score)	1.04 (0.99,1.09)	0.164	1.04 (0.99,1.09)	0.155	1.03 (0.98,1.09)	0.180	1.05 (0.99,1.10)	0.082	1.04 (0.99,1.09)	0.145
Residential density (z-score)*	1.03 (0.91,1.17)	0.605	1.03 (0.91,1.16)	0.618	1.04 (0.92,1.17)	0.570	1.03 (0.91,1.16)	0.660	1.03 (0.92,1.16)	0.594
Connectivity (z-score)*	1.06 (0.94,1.19)	0.337	1.06 (0.94,1.19)	0.336	1.06 (0.94,1.19)	0.339	1.06 (0.94,1.19)	0.372	1.06 (0.94,1.19)	0.357
Land use mix (z-score)*	1.01 (0.91,1.14)	0.801	1.02 (0.91,1.14)	0.747	1.01 (0.90,1.13)	0.900	1.06 (0.95,1.18)	0.328	1.02 (0.91,1.14)	0.711

OR = odds ratio; CI = confidence interval; *Denotes multivariate association; ^a ^Model 1 = Residential+Retail+Office+Health, welfare & community; ^b ^Model 2 = Model 1+Entertainment, culture & recreation; ^c ^Model 3 = Model 2+Public Open Space, Sporting infrastructure & Primary and rural; ^d ^Model 4 = Model 3+Unclassified land; ^e ^Model 5 = Residential+Retail+Health, welfare & community+Entertainment, culture & recreation+Public open space, Sporting infrastructure & Primary and rural. All models adjusted for gender, age, education level, marital status and presence of children at home.

Overall there were no significant associations between the WI and total walking or recreational walking ≥ 150 mins/week across all models (Table 6). However, a significant continuous trend test for the WI and ≥ 150 mins/week of transport walking was found in Models 1, 2 and 4.

## Discussion

The aim of this study was to explore how variations in the categories of land uses included in entropy calculations of LUM measures in the WI can impact on the observed associations with total, transport and recreational walking. This study is unique in that it allowed a comparison of different LUM computations within the same data set. Until now comparisons have only been possible between different LUM measures used in different studies in different contexts and this limits the comparability of findings.

Irrespective of the LUM measure used, our results show that residents living in high walkable neighbourhoods do more walking than those in low walkable environments and that WIs are more strongly related to walking for transport than recreational walking. Depending upon what LUM was incorporated into the WI, residents living in highly walkable neighbourhoods were up to twice as likely to walk for transport as residents in low walkable neighbourhoods. While these findings agree with the work of others [15], our results show that the associations varied by type of walking, and by the *amount *of walking (e.g., > 0, ≥ 60 or ≥ 150 mins/week). Owen et al., also reported differences by type of walking with significant associations with walking for transport but no association with recreational walking [19]. Our findings show that reporting more than an hour per week of transport walking had the strongest and most significant association with a WI that included 'Residential', 'Retail', 'Office', 'Health, welfare and community', *and *'Entertainment, culture and recreation', while doing any recreational walking was more strongly associated with a WI that also included 'Public open space', 'Sporting infrastructure' and 'Primary and rural' land uses. There was no association with higher levels of walking (≥ 150 mins/week) however the prevalence of respondents achieving this level of neighbourhood walking was low and this may have reduced the power to detect significant associations. The variations observed lend further support to the idea that context-specific measures of the built environment (e.g., a recreational walking specific WI) would be more sensitive to detecting associations with different types of walking behaviour [25,31,43,44].

Importantly, this study provides evidence that varying the combination of land uses in the LUM calculation impacts the strength of relationships with different types (and amounts) of walking behaviour. The strongest association between the WI and any transport walking was found in the land use mix computation that included 'Residential', 'Retail', 'Office', 'Health, welfare and community' and 'Entertainment, culture and recreation' land use classifications (Model 2). This LUM is most similar to the later computations of WIs used by Frank and colleagues [16]. In contrast, Model 3, which included Model 2 land classifications plus 'Public open space', 'Sporting infrastructure' and 'Primary and rural', better captured recreational walking. The construction of Model 3 was based upon the work of Forsyth et al., [41]. Notably, when 'Public open space', 'Sporting infrastructure' and 'Primary and rural' was included in the LUM measure (Model 3), the association between the land use z-score and transport walking was eliminated confirming that a land use class that includes public open space, is not relevant for transport walking. Rather a LUM that incorporates transport-related destinations only (i.e., Model 2) appears to be superior for capturing an association between walkability and transport walking. Similarly, Duncan et al., [24] reported that the relationship between Census Collector District-level LUM and walking for transport is stronger when using LUM measures that include only theoretically relevant land uses. These results support our hypothesis that different computations of land use mix are relevant for different types of walking. Furthermore, the results are promising in that they provide evidence to suggest that manipulation of land uses included in the LUM measure can result in improved associations with recreational walking. This work provides an important first step towards developing a WI that better captures recreational walking although further work is required.

While the aim of this study was to manipulate land use classes to best capture walking behaviours, there are inherent problems with the base data and the calculation used to determine LUM (i.e., entropy formulas) which present significant barriers to the development of behaviour-specific LUM measures. It has already been highlighted [41] that to fully understand the results from these kinds of analyses, a detailed knowledge of the base data is important, particularly the data from which the land use classes are derived. Similar to other studies of this kind, the land classification system used in RESIDE was designed for planning purposes and commercial employment patterns [39], not public health research. Various data processing steps are therefore required to create land use measures and these steps are often restricted by the original base data structure and coding. Moreover, data processing can be undertaken in different ways which may not be clearly reported when published. Often the preferred specificity and groupings of land uses are not available or possible from the base data and this could impact on the relationships detected. At worst, the use of broad groupings of land use may obscure associations between the environment and behaviour of interest. These limitations have been observed previously in ecological studies of plant and animal distributions [45].

Another problem with the base data used to compute the LUM variable is the allocation of a single use to a land area when, in some instances, a multi-use classification may be more appropriate. For example, a large city park with a small kiosk on site would be classified as a large 'Retail' area based on the single-use hierarchy of land use classifications in Table 1. Not only does this classification fail to represent the reality on the ground (i.e., presence of both green space and a retail outlet) but it would likely alter the observed associations between the neighbourhood attributes and specific-walking behaviours. A more appropriate classification for this land parcel would have been both 'Public open space' and 'Retail' classifications.

A further limitation associated with base data is incomplete data coverage. Land uses may be omitted from the spatial classification system due to insufficient data. For example, in RESIDE it was likely that areas identified as 'Unclassified' included attractive vegetation and/or natural amenities such as waterways (streams) conducive to recreational walking. Thus, exclusion of unclassified land in the base data set may have attenuated associations with recreational walking. We therefore tested models with (Model 4) and without (Model 5) the 'Unclassified' land use to explore its potential contribution but found when added there was no association with recreational walking. We suggest that it is possible that the 'unclassified' category may include land uses that are both positively (vegetation) and negatively (derelict land) associated with walking. Future studies may therefore wish to explore this further, but in the interim it appears justified within the West Australian context to exclude this land classification and remove the 'noise' associated with potential measurement error.

Another underlying issue potentially affecting the observed relationships is the calculation of LUM itself, specifically the limitations associated with the entropy formula. As highlighted by Brown and colleagues [28] in a study exploring patterns of obesity, entropy scores of LUM have a number of limitations and these include: not capturing the presence of a wide range of land uses (usually only a maximum of six land use classes included); each land use class is treated as equal when the relationship between different land uses may be relative to one another; not capturing differences in the aesthetic appeal of land uses; and as noted above, unclassified land is simply ignored. Furthermore, entropy scores give a relative score of land use (range 0-1) and do not reflect the absolute size of area. Despite these limitations, the RESIDE study had access to a reasonably well organised and accessible source of land use data. Unlike studies that report a lack of coordination in the collection of land use information [46], information from the Values General's Office of Western Australia and the public land vesting information [37,40] provide a strong data infrastructure that can be manipulated to support public health research. However, the methodological issues noted here, highlight that comparisons between studies may be problematic and caution is required when undertaking within-and between-country comparisons of the association between neighbourhood walkability and physical activity.

It is evident that the prediction of different amounts and types of walking behaviours may depend on the types and combinations of land use classes included in the LUM component of a WI. A simple measure of the total area of 'walkable' land uses (e.g., public open space, retail, residential) may provide a better measure of LUM than an entropy score. For example, Brown and colleagues reported that for body mass index the presence of walkable land uses was more important than the equal mix of walkable land uses calculated from entropy scores [28]. Furthermore, the presence or density of specific destinations is relatively easy to compute and is viewed as an acceptable substitute for LUM measures [47-49]. Nevertheless, it can be difficult to generate a concise and current listing of destinations in a study area and considerable variation in data quality exists between commercially available sources and researcher-conducted field audit data [50]. Until this issue can be resolved, the use of destination data in WIs may be limited.

It is also possible that other attributes of urban design over and above LUM may improve the explanatory value of WIs. For example, the presence or absence of sidewalks, the amount of natural vegetation (greenness index), road traffic volume, as well as the aesthetic quality of the neighbourhood could be included in an expanded WI. This may result in stronger associations with walking behaviours, which may vary across the life course from children through to older adults. Others have noted that WIs that do not include measures of aesthetics may contribute to the failure to predict variation in patterns of recreational walking [23,31]. Future RESIDE analyses will investigate ways to create neighbourhood walkability measures which have a stronger relationship with recreational walking. As a longitudinal cohort study RESIDE is also uniquely placed to explore associations over time to determine if changes in neighbourhood walkability causes people to do more or less transport and recreational walking.

Finally, it is possible that the association between different types of walking and LUM and other design characteristics could vary by different scale [24,51,52]. RESIDE used a 1600 m service area to define a person's neighbourhood because theoretically, it represents how far a participant could walk from their house at 'moderate' intensity pace within 15 minutes, half the recommended daily physical activity for an adult [53]. Future research should explore variations in LUM computations at different scales and consider the use of Global Positioning Systems (GPS) units to examine variation in both size and shape of participant's neighbourhoods and the effect this has on the association between LUM and walking behaviour. Another area of future research could involve examining thresholds for the components of WIs in different areas. At this stage, cut points for WI quartiles are sample-specific. To enable study comparisons, pooled data from different areas would enable cut points to be established.

### Study limitations

Although RESIDE is a quasi experimental study, the data presented are cross-sectional and causality cannot be inferred. A number of GIS-related limitations mentioned in the discussion are relevant to this study. The land use base data may not accurately represent what is actually present in the environment and was not assessed for its accuracy, which is a limitation. Furthermore, the allocation of land use to a single use prevents multi-use classifications and this could have resulted in LUM scores for some neighbourhoods being under-represented. Moreover, as RESIDE participants are people building new homes, they are not representative of the general population. As they were selected from people building homes across the entire metropolitan area, they are however, likely to be representative of new home buyers. In low density car dependent cities seen in Australia and the US, walking and cycling are likely to make a smaller contribution to total physical activity compared with (say) Europe. This will limit the associations observed, and thus there is a need for global thresholds of components of WIs to enable comparisons across countries. Finally, the limitations of using self-report physical activity data are well documented [54].

## Conclusions

Overall our findings provide further evidence that participants' in high versus low walkable neighbourhoods report more walking and that WIs are most strongly associated with transport walking. Varying the combination of land uses in the LUM calculation of WIs affects the strength of relationships with different types (and amounts) of walking. Reporting more than an hour per week of transport walking had the strongest and most significant association with a WI that included 'Residential', 'Retail', 'Office', 'Health, welfare and community', *and *'Entertainment, culture and recreation', while doing any recreational walking was more strongly associated with a WI that also included 'Public open space', 'Sporting infrastructure' and 'Primary and rural' land uses. This study is the first to show, within the same data set, the association between walking and WI computations that use different land use categories and provides an important first step towards developing a WI that better captures recreational walking. However, inherent problems with the base data and the use of entropy formulas may be restricting this field of research. Notable issues include incomplete data coverage, single allocation of land use classification, and aggregated land use categories providing insufficient specificity. The development of GIS measures would be aided if there was more transparency and clarity on the sourcing, handling and limitations of base data and development of LUM calculations. Further, alternate or complementary methods to entropy formula for calculating LUM should be considered (such as total area of 'walkable' land uses or density and types of walkable destinations). The development of WIs specific to different types of physical activity, including walking for different purposes, is likely to require inclusion of other attributes of the environment such as footpaths, traffic volume, safety and these expanded models should be explored.

## Competing interests

The authors declare that they have no competing interests.

## Authors' contributions

All authors contributed to the study conception and design, interpretation of results and provided input to manuscript drafts. HC also interpreted the data, results and implications of the study and drafted the manuscript revising it critically at each stage. MK advised on the data analysis and interpretation of results. MD and AA conducted the analyses. NM and FB provided input on the implications of the study and assisted the first author with drafting the manuscript. All authors read and approved the final manuscript.
